# Clinical features, spectrum of causal genetic mutations and outcome of hypertrophic cardiomyopathy in South Africans

**DOI:** 10.5830/CVJA-2015-075

**Published:** 2016

**Authors:** Ntobeko AB Ntusi, Gasnat Shaboodien, Motasim Badri, Bongani M Mayosi, Motasim Badri, Freedom Gumedze

**Affiliations:** Cardiovascular Genetics Laboratory, Hatter Institute for Cardiovascular Research in Africa and The Cardiac Clinic, Department of Medicine, Groote Schuur Hospital and University of Cape Town, Cape Town, South Africa; Cardiovascular Genetics Laboratory, Hatter Institute for Cardiovascular Research in Africa and The Cardiac Clinic, Department of Medicine, Groote Schuur Hospital and University of Cape Town, Cape Town, South Africa; Cardiovascular Genetics Laboratory, Hatter Institute for Cardiovascular Research in Africa and The Cardiac Clinic, Department of Medicine, Groote Schuur Hospital and University of Cape Town, Cape Town, South Africa; Cardiovascular Genetics Laboratory, Hatter Institute for Cardiovascular Research in Africa and The Cardiac Clinic, Department of Medicine, Groote Schuur Hospital and University of Cape Town, Cape Town, South Africa; King Saud Bin Abdulaziz University for Medical Sciences, Riyadh, Kingdom of Saudi Arabia; Department of Statistical Sciences, University of Cape Town, Cape Town, South Africa

**Keywords:** hypertrophic cardiomyopathy, genetics, clinical characteristics, outcome, South Africa

## Abstract

**Background:**

Little is known about the clinical characteristics, spectrum of causal genetic mutations and outcome of hypertrophic cardiomyopathy (HCM) in Africans. The objective of this study was to delineate the clinical and genetic features and outcome of HCM in African patients.

**Methods:**

Information on clinical presentation, electrocardiographic and echocardiographic findings, and outcome of cases with HCM was collected from the Cardiac Clinic at Groote Schuur Hospital over a mean duration of follow up of 9.1 ± 3.4 years. Genomic DNA was screened for mutations in 15 genes that cause HCM, i.e. cardiac myosinbinding protein C (MYBPC3), cardiac β-myosin heavy chain (MYH7), cardiac troponin T2 (TNNT2), cardiac troponin I (TNNI3), regulatory light chain of myosin (MYL2), essential light chain of myosin (MYL3), tropomyosin 1 (TPM1), phospholamban (PLN), α-actin (ACTC1), cysteine and glycine-rich protein 3 (CSRP3), AMP-activated protein kinase (PRKAG2), α-galactosidase (GLA), four-and-a-half LIM domains 1 (FHL1), lamin A/C (LMNA) and lysosomeassociated membrane protein 2 (LAMP2). Survival and its predictors were analysed using the Kaplan–Meier and Cox proportional hazards regression methods, respectively.

**Results:**

Forty-three consecutive patients [mean age 38.5 ± 14.3 years; 25 (58.1%) male; and 13 (30.2%) black African] were prospectively enrolled in the study from January 1996 to December 2012. Clinical presentation was similar to that reported in other studies. The South African founder mutations that cause HCM were not found in the 42 probands. Ten of 35 index cases (28.6%) tested for mutations in 15 genes had disease-causing mutations in MYH7 (six cases or 60%) and MYBPC3 (four cases or 40%). No disease-causing mutation was found in the other 13 genes screened. The annual mortality rate was 2.9% per annum and overall survival was 74% at 10 years, which was similar to the general South African population. Cox’s proportional hazards regression showed that survival was predicted by New York Heart Association (NYHA) functional class at last visit (*p* = 0.026), but not by the presence of a disease-causing mutation (*p* = 0.474).

**Conclusions:**

Comprehensive genetic screening was associated with a 29% yield of causal genetic mutations in South African HCM cases, all in MYH7 and MBPC3 genes. A quarter of the patients had died after a decade of follow up, with NYHA functional class serving as a predictor of survival.

## Background

Hypertrophic cardiomyopathy (HCM) is defined by the presence of myocardial hypertrophy in the absence of haemodynamic stresses sufficient to account for the degree of hypertrophy (e.g. arterial hypertension and aortic stenosis), and without other secondary causes of cardiac hypertrophy, such as amyloidosis and glycogen storage disease.^1^

HCM was historically thought to be rare among Africans.^2^ This impression was reinforced by a study that found HCM to occur in 0.2% of 6 680 unselected echocardiograms performed in Tanzania.^3^ However, recent echocardiographic studies from the continent have dispelled that myth.^4^ For example, in Ghana, HCM has been reported to be the third commonest cardiomyopathy after dilated cardiomyopathy (DCM) and endomyocardial fibrosis (EMF).^5^ Similarly, in Ethiopia, HCM accounts for 34% of all cardiomyopathies diagnosed on echocardiography.^6^ However, there is a dearth of information on the clinical features, genetics and outcome of HCM from the African continent, with a few publications reporting on HCM-causing mutations in South Africans of northern European descent and mixed ancestry.^7^-^10^ To the best of our knowledge, there are no data on the genetics of HCM in black Africans.

HCM is a diverse disease with variable phenotypic expression; a substantial proportion of patients live a normal life with minimal risk of sudden cardiac death.^11^ However, some patients with or without symptoms may die suddenly, even without having clinical features of severe left ventricular hypertrophy (LVH).^9^

The pattern of LVH in HCM is variable and associated with differences in morbidity and mortality. For instance, apical HCM in the Japanese and North American populations is associated with a benign outcome.^12^ The clinical pattern and outcome of HCM in Africans is not known. The aim of this study was to delineate the clinical features, spectrum of diseasecausing mutations and outcome of HCM in African patients.

## Methods

Consecutive patients diagnosed with HCM at the Cardiac Clinic, Groote Schuur Hospital (GSH), Cape Town, South Africa were prospectively enrolled into a longitudinal cohort study of familial cardiomyopathy, from 1 February 1996 to 31 August 2012. The diagnosis of HCM was based on the presence of a hypertrophied, non-dilated left ventricle in the absence of other diseases capable of producing the degree of observed LVH (i.e. left ventricular wall thickness > 14 mm on echocardiography).^13^ Clinical data were collected at six-monthly visits during the study period.

The study was designed in keeping with the principles of the Helsinki Declaration, and was approved by the University of Cape Town Human Research Ethics Committee. All participants gave informed, written consent to participate in the study.

All patients had comprehensive clinical assessment, complemented by chest radiography, electrocardiography, detailed two-dimensional and Doppler colour-flow echocardiography, and cardiac catheterisation, when appropriate. The primary imaging modality used for diagnosis in all patients was transthoracic two-dimensional and Doppler echocardiography. Patients found to have outflow tract gradients below 40 mmHg underwent Valsalva manoeuvre. Patients with cardiovascular risk factors, angina or subjects over 40 years old frequently underwent coronary angiography, at the discretion of the attending clinician.

A comprehensive database that incorporated patient demographic details, medical history, co-morbidity, medical therapy, clinical, electrographic and echocardiographic details was utilised. Normal values for echocardiographic measurement were based on age and body-surface area, as described by Lauer *et al*.14

## Genotyping

Peripheral blood was collected from HCM probands for DNA extraction using standard methods. Mutation screening was undertaken by pyrosequencing of the coding regions and exon/ intron boundaries of the following 15 genes that are associated with HCM: cardiac myosin-binding protein C (MYBPC3), cardiac β-myosin heavy chain (MYH7), cardiac troponin T type 2 (TNNT2), cardiac troponin I type 3 (TNNI3), regulatory light chain of myosin (MYL2), essential light chain of myosin (MYL3), tropomyosin 1 (TPM1), phospholamban (PLN), α-actin (ACTC1), cysteine and glycine-rich protein 3 (CSRP3), AMP-activated protein kinase (PRKAG2), α-galactosidase (GLA), four-and-a-half LIM domains 1 (FHL1), lamin A/C (LMNA) and lysosome-associated membrane protein 2 (LAMP2) (Table 1).^15^

**Table 1:** List of genes that were subjected to mutation screening in this study

*Genes*	*Ensemble gene number*	*Chromosome: base range*
MYBPC3	ENSG00000134571	chr11:47352958–47374253
MYH7	ENSG00000092054	chr14:23881948–23904870
TNNT2	ENSG00000118194	chr1:201328143–201346805
TNNI3	ENSG00000129991	chr19:55663137–55669100
TPM1	ENSG00000140416	chr15:63334838–63364111
MYL2	ENSG00000111245	chr12:111348626–111358404
MYL3	ENSG00000160808	chr3:46899357–46904973
ACTC1	ENSG00000159251	chr15:35080297–35087927
PLN	ENSG00000198523	chr6:118869442–118881586
CSRP3	ENSG00000129170	chr11:19203578–19223589
FHL1	ENSG00000022267	chr X:135229559-135293518:1
PRKAG2	ENSG00000106617	chr 7:151253197-151574210
GLA	ENSG00000102393	X:100652791-100662913
LMNA	ENSG00000160789	chr1:156084461–156109878
LAMP2	ENSG00000005893	chr X:119561682-119603220

MYBPC3, myosin-binding protein 3; MYH7, beta-myosin heavy chain; TNNT2, troponin T; TNNI3, troponin I; MYL2, essential myosin light chain; MYL3, regulatory myosin light chain; TPM1, tropomyosin 1 alpha; PLN, phospholamban; ACTC1, actin; CSRP3, cysteine- and glycine-rich protein 3; PRKAG2, 5′-AMP-actvated protein kinase; GLA, alpha-galactosidase; FHL1, four-and-a-half LIM domains 1; LMNA, lamin A/C; LAMP2, lysosome-associated membrane protein.

Exons and intron/exon boundaries (± 10 base pairs) of the 15 cardiomyopathy-related genes were amplified by microdroplet polymerase chain reaction (PCR) using RDT 1000 technology (Rain Dance Technologies, Billerica, MA 01821, USA). Libraries were prepared using the Rapid Library 454 FLX protocol, which included adding molecular identifiers to each sample. Samples were pooled and then sequenced using the Roche 454 FLX nextgeneration sequencing platform. Samples were processed and analysed using NextGENe version 2.2.0 (SoftGenetics).

Prior to analysis, reads were trimmed and low-quality reads were removed. Reads were aligned to .gbk files and variants seen in < 20% were annotated. Variants were filtered, taking into account coverage, read balance, allele balance and homopolymers. Samples with coverage below 10 were considered failures. Unclassified variants were Sanger sequenced to confirm their presence; known polymorphisms were not Sanger sequenced.

The Cape Town population controls were used to determine the population frequencies of all novel variants identified in the 15 genes. One hundred and ninety-five anonymous blood donors from the Western Province Blood Transfusion Service provided consent for blood samples to be taken for DNA extraction. The control DNA consisted of samples from 95 persons of mixed ancestry, 50 black Africans and 50 white South Africans.

## Statistical analysis

Simple descriptive statistics were used for data interpretation and to draw inferences about the population of patients studied. Results of continuous variables are given as means ± SD. Categorical variables are represented as number and percentage. Pearson’s chi-squared or Fisher’s exact test were used to compare the relative frequency of characteristics between individuals. All *p*-values were two-sided, and *p* ≥ 0.05 was considered not to indicate statistical significance.

Survival analysis testing between groups was compared using log-rank testing, and the Kaplan–Meier survival curves were constructed using the product-limit method. Age-, gender- and race-adjusted survival curves for the general South African population were derived and compared with the Kaplan–Meier survival rates for the patients with HCM. Analysis included univariate and multivariate regression analysis, with a focus on mortality rather than time to death, therefore justifying the use of Cox’s proportional hazards model rather than logistic regression analysis.

## Results

The study cohort comprised 43 patients with HCM. The clinical characteristics and co-morbid status of the study population at the initial evaluation are shown in Table 2. The mean age of HCM patients studied was 38.5 ± 14.3 years; 25 (58.1%) were male. Thirteen (30.2%) were black Africans, and the majority (62.8%) were of mixed ancestry. Twenty-six (60.5%) had firstdegree relatives with HCM and five (11.6%) had a family history of sudden cardiac death (SCD) in a first-degree relative. Symptoms of palpitations (79.1%), angina (65.1%), fatigue (58.1%) and effort-related breathlessness (55.8%) were frequently reported by the patients. Ten (23.3%) had a New York Heart Association (NYHA) functional capacity of class III at the initial assessment. An ejection systolic murmur was reported in 18 (41.9%) of patients.

**Table 2:** Demographic, clinical, electrocardiographic and echocardiographic features at presentation in patients with hypertrophic cardiomyopathy compared to three large contemporary international reports from North America, Taiwan and Saudi Arabia

*Medical history*	*South Africa (n = 43)*	*North America (n = 277)*	*Taiwan (n = 163)*	*Saudi Arabia (n = 69)*
Age at diagnosis (years)	38.5 ± 14.3	47 ± 22	60.9 ± 12.1	42 ± 16
Males	25 (58)	152 (55)	84 (52)	43 (71)
Ethnicity (%)				
Black/African	13 (30)			
White/Caucasian	2 (5)	277 (100)		
Coloured/mixed ancestry	27 (63)			
Indian ancestry	1 (2)			
Taiwanese			163 (100)	
Arab				69 (100)
First-degree relative with HCM	26 (61)	21 (8)	–	2 (5)
Second-degree relative with HCM	7 (16)	–	–	–
Has family history of SCD	5 (12)	–	–	4 (9)
NYHA functional class				
I and II	33 (77)	–	–	–
III and IV	10 (23)			
Symptoms		174 (63)		
Fatigue	25 (58)		–	–
Dyspnoea	24 (56)		121 (74)	31 (65)
Palpitations	34 (79)		28 (17)	5 (7)
Angina	28 (65)		111 (68)	–
Presyncope/syncope	12 (28)		20 (12)	2 (4)
Smoking	19 (31)	–	–	–
Hypertension	12 (28)	–	28 (17)	–
Diabetes	0 (0)	–	29 (18)	–
Alcohol consumption	9 (21)	–	–	–
Dyslipidaemia	6 (14)	–	–	–
Coronary artery disease	3 (7)	–	29 (18)	–
COPD	2 (5)	–	–	–
HIV infection	2 (5)	–	–	–
Medical examination				
Heart rate	71.3 ± 12.7	–	–	–
BP_sys_	125.8 ± 19.2	–	–	–
BP_dia_	75.8 ± 11.3	–	–	–
Pedal oedema	5 (11.6)	–	–	–
Ejection systolic murmur	18 (41.9)	–	–	–
Electrocardiographic findings				
Sinus rhythm	39 (90.7)	–	–	–
Atrial fibrillation	4 (9.3)	–	34 (21)	–
QRS abnormalities present	12 (28)	–	–	–
Voltage criteria for LVH	22 (51)	–	137 (84)	60 (87)
Presence of pathological Q waves	12 (28)	–	–	–
T-wave inversion	34 (79)	–	108 (66)	–
Left atrial hypertrophy	10 (23)	–	–	–
LBBB	4 (9)	–	–	–
RBBB	2 (5)	–	–	–
PR prolongation	2 (5)	–	–	–
Echocardiographic findings				
LVEDD (cm)	4.1 ± 0.8	–	4.5 ± 0.5	–
LVESD (cm)	2.7 ± 0.6	–	2.4 ± 0.4	–
IVS_dia_ (cm)	1.9 ± 0.7	2.2*	1.9 ± 0.4	–2.1 ± 0.7
IVS_sys_ (cm)	2.1 ± 0.7	–	–	–
LVPFW_dia_ (cm)	1.2 ± 0.4	-	1.1 ± 0.3	1.3 ± 0.4
LVEF (%)	71.5 ± 8.3	-	-	68 ± 13
Left atrial size (cm)	3.5 ± 0.8	-	3.8 ± 0.7	-
SAM	9 (21)	-	80 (49)	39 (57)
LVOT obstruction	12 (28)	-	78 (48)	28 (41)
E/A ratio	1.2 ± 0.4	-	-	1.5 (0.9–2.1)
Pattern of hypertrophy				
Sigmoid	13 (30)	75 (27)	-	8 (12)
Catenoid	23 (53)	92 (33)		29 (42)
Neutral	6 (14)	65 (23)		25 (36)
Apical	1 (2)	5 (2)		7 (10)

All results are means ± standard deviation, unless otherwise indicated.

*No standard deviation given.

BP_dia_, diastolic blood pressure; BP_sys_, systolic blood pressure; COPD, chronic obstructive pulmonary disease; E/A, ratio of early (E) to late (A) ventricular filling velocities on Doppler echocardiography; IVS_dia_, interventricular septal thickness in diastole;IVS_sys_, interventricular septal thickness in systole; HCM, hypertrophic cardiomyopathy; HIV, human immunodeficiency virus; LBBB, left bundle brunch block morphology on electrocardiography; LVEDD, left ventricular end-diastolic dimension; LVEF, left ventricular ejection fraction; LVESD, left ventricular end-systolic dimension; LVH, left ventricular hypertrophy; LVPFW_dia_, left ventricular posterior free-wall thickness in diastole; LVOT, left ventricular outflow tract; NYHA, New York Heart Association functional classification for severity of breathlessness; RBBB, right bundle brunch block morphology on electrocardiography; SAM, systolic anterior motion of the anterior mitral valve leaflet; SCD, sudden cardiac death.

The electrocardiographic and echocardiographic characteristics of the study population are shown in Table 2. Four (9.3%) of the HCM patients had atrial fibrillation at diagnosis. On echocardiography, the mean left ventricular (LV) septal thickness in diastole, LV ejection fraction (LVEF) and left atrial diameter were 1.9 ± 0.7 cm, 71.5 ± 8.3% and 3.5 ± 0.8 cm, respectively. Left ventricular outflow tract (LVOT) obstruction, with a resting gradient of greater than 10 mmHg was found in 12 (27.9%) patients. Evidence of diastolic dysfunction was present in the majority of patients, and the mean E/A ratio was 1.2 ± 0.4.

## Spectrum of mutations that cause HCM in South Africans

Of the 43 patients diagnosed with HCM, 42 were screened for the common founder mutations previously described in the South African population, and all 42 were found to be negative for these variants.^10^ Further molecular genotypic analysis was undertaken in 35 of these HCM patients for 15 cardiomyopathy-associated genes. Of these 35 probands, mutation screening yielded disease-causing mutations in 10 unrelated individuals (28.6%) (Table 3). The disease-causing mutations were found in two out of the 15 genes screened, with the majority in MYH7 (*n* = 6 probands; 60%) and the rest in MYBPC3 (*n* = 4 probands; 40%). Two of the MYH7 mutations were novel, and diseasecausing mutations were found in all ethnic groups tested. No single disease-causing mutation occurred in more than one study subject.

**Table 3:** Disease-causing mutations found in 10 unrelated index cases with hypertrophic cardiomyopathy

*Index case ID*	*Ethnicity*	*Reported previously?*	*Gene*	*Exon*	*Nucleotide and amino acid change*	*Type of mutation*	*Reference*
HCM1.1	Indian	Yes	MYH7	5	c.611G>A (p.R204H)	Missense	Richard, et al. 2003^18^
HCM4.1	Mixed ancestry	No	MYH7	20	c.2282C>A (p.T761N)	Missense	Novel
HCM7.1	Mixed ancestry	Yes	MYH7	31	c.4258C>T (p.R1420W)	Missense	Zou, et al. 2013^22^
HCM11.1	Mixed ancestry	Yes	MYBPC3	12	c.1000G>A (p.E334K)	Missense	Bahrudin, et al. 2008^23^
HCM14.1	European	No	MYH7	20	c.2167C>T (p.R723C)	Missense	Novel
HCM16.1	European	Yes	MYH7	9	c.746G>A (p.R249Q)	Missense	Zou, et al. 2013^22^
HCM21.1	Black African	Yes	MYBPC3	6	c.772G>A (p.E258K)	Missense	Andersen, et al. 2004^24^
HCM33.1	Mixed ancestry	Yes	MYBPC3	5	c.530G>A (p.R177H)	Missense	University of Stellenbosch thesis^25^
HCM34.1	Black African	Yes	MYH7	14	c.1357C>T (p.R453C)	Missense	Zou, et al. 2013^22^
HCM38.1	Black African	Yes	MYBPC3	15	c.1246G>A (p.G416S)	Missense	Tanjore, et al. 2008^26^

There were three genetic variants of unknown significance found in *MYBPC3*, which were not observed in 195 population controls: c.1224-19G>A, c.1790+5G>A, and c.133G>A. In addition, a large number of known polymorphisms were found in 16 probands in *MYBPC3* (tmp_esp_11_47355301, rs113941605 and rs113658284), *MYH7* (rs149439730, rs45523835, rs145738465, rs202205780, rs61737803, rs146858930, rs36211714, rs45501694, and rs111626355), *TNNT2* (rs113471285, and rs115805892), *TPM1* (tmp_esp_15_63356347), *MYL3* (rs199474709), *CSRP3* (rs112848043), *FHL1* (rs182106777), *PRKAG2* (rs116605521, and rs113234987), *GLA* (rs151195362), and *LMNA* (rs12117552, and rs117939448). Two novel single-nucleotide polymorphisms were found in *MYH7* (c.1368C>T) and *PRKAG2* (c.828C>A) in two different probands.

The total number of variants in the 15 genes per HCM patients (regardless of whether it was disease causing or not) ranged from six to 20 (mean: 12.8, SD 3.2; median 12). The patient who died had a higher number of variants (14.8 ± 3.9) compared to survivors (12.5 ± 3.1), although this difference was not statistically significant (*p* = 0.47).

## Outcome of HCM in South Africans

**Fig. 1.**
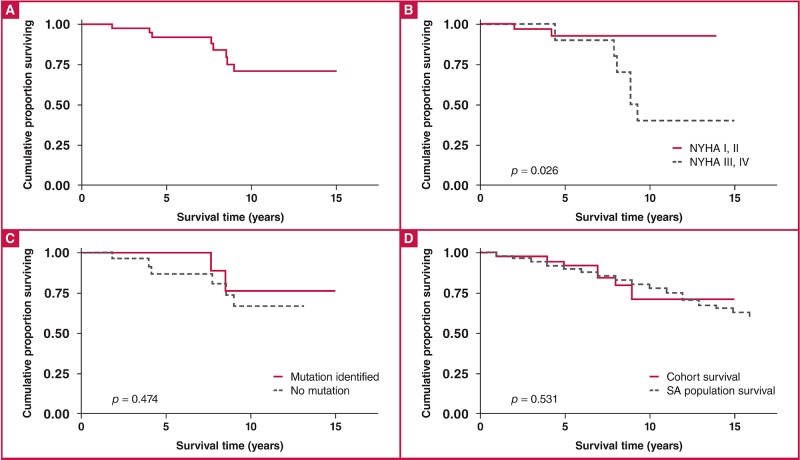
The process of Confidential Enquiry into Maternal Deaths.

The mean duration of follow up was 9.1 ± 3.4 years. Of the 43 patients studied, eight died during the period of follow up. The overall Kaplan–Meier survival estimate is shown in Fig. 1A; the cumulative proportion of patients who survived to 10 years was 74%. Complications of chronic heart failure, atrial fibrillation, stroke and evolution to dilated cardiomyopathy with systolic dysfunction were observed in 11 (25.6%), eight (18.6%), four (9.3%) and four (9.3%), respectively.

Therapeutic interventions, including surgical myomectomy, alcohol septal ablation and orthotopic heart transplantation were performed on three (7.0%), one (2.3%) and one (2.3%) patients, respectively. At the last visit, 12 (27.9%) reported NYHA functional class III and IV performance status (Table 4). The most frequently prescribed drugs were beta-blockers and calcium channel blockers, used by 33 (76.7%) and 17 (39.5%) patients, respectively (Table 5).

**Table 4:** Follow-up and outcome data

*Outcome data*	*n (%)*
Mean duration of follow up (years ± SD)	9.1 ± 3.4
Total number of mutations per person	12.8 (3.2)
Follow-up observation	
Regular	37 (86.0)
Lost to follow up	6 (14.0)
Death	8 (18.6)
Chronic heart failure	11 (25.6)
ICD insertion	0 (0)
PPM insertion	0 (0)
CRT/biventricular pacing	0 (0)
Loop recorder	6 (14.0)
Arrhythmia present	
No arrhythmia	32 (74.4)
Atrial fibrillation	8 (18.6)
Atrial flutter	1 (2.3)
Ventricular tachycardia	2 (4.7)
Myomectomy	3 (7.0)
Alcohol septal ablation	1 (2.3)
Evolution to DCM	4 (9.3)
Orthotopic heart transplantation	1 (2.3)
NYHA functional class at last visit	
I and II	31 (72.1)
III and IV	12 (27.9)
Stroke	4 (9.3)

All values are number (percentage), unless otherwise stated.

ICD, implantable cardioverter defibrillator; PPM, permanent pacemaker; CRT, cardiac resynchronisation therapy; DCM, dilated cardiomyopathy; NYHA, New York Heart Association functional classification for evaluation of severity of dyspnoea.

**Table 5:** Medical therapy at follow up

*Therapy*	*n (%)*
β-blocker	33 (76.7)
Calcium channel blocker	17 (39.5)
Warfarin	12 (27.9)
ACEI or ARB	9 (20.9)
Furosemide	8 (18.6)
Aspirin	8 (18.6)
Disopyramide	4 (9.3)
Spironolactone	4 (9.3)
Amiodarone	3 (7.0)
Digoxin	2 (4.7)
Nitrates	1 (2.3)

Cox’s proportional hazards regression showed that survival was predicted by NYHA functional class at last visit (p = 0.026), but not by presence of a disease-causing mutation (p = 0.474), as shown in Figs 1B and 1C, respectively. Survival in this cohort was similar to that of an age- and gender-matched general South African population (Fig. 1D).

The presence of chronic heart failure [hazard ratio (HR) 4.4, 95% CI: 1.0–18.3; p = 0.044] and NYHA functional class at last visit (HR: 6.2, 95% CI: 1.2–30.6; *p* = 0.026) were found to be predictors of mortality on univariate regression analysis. On multivariate analysis, both chronic heart failure and NYHA functional class were not significant as predictors of mortality, as they may be proxies for LVEF (Table 6).

**Table 6:** Cox’s proportional hazards regression model analysis of predictors of mortality in hypertrophic cardiomyopathy)

	*Univariate Cox regression*	
*Variables*	*Hazard ratio (95% CI)*	*p-value*
Age at diagnosis	1.0 (1.0–1.1)	0.561
Mutation positive	1.8 (0.4–8.9)	0.474
Sarcomeric mutations	1.3 (0.5–3.5)	0.585
Total number of mutations per person	1.12 (0.91–1.3)	0.412
IVS	1.6 (0.8–3.4)	0.169
LVEF	1.1 (1.0–1.2)	0.060
Family history of SCD	0.8 (0.1–6.6)	0.840
E/A ratio	2.0 (0.4–10.0)	0.370
Loop recorder	3.5 (0.8–14.6)	0.088
Chronic heart failure	4.4 (1.0–18.3)	0.044
NYHA functional class at last visit	6.2 (1.2–30.6)	0.026
	*Multivariate Cox regression*	
*Variables*	*Hazard ratio (95% CI)*	*p-value*
LVEF	1.1 (1.0–1.2)	0.100
Loop recorder	0.8 (0.1–5.3)	0.828
Chronic heart failure	1.6 (0.2–16.2)	0.684
NYHA functional class at last visit	4.2 (0.4–41.3)	0.218

E/A, ratio of early (E) to late (A) ventricular filling velocities on Doppler echocardiography; IVS, interventricular septal thickness in diastole; HCM, hypertrophic cardiomyopathy; LVEF, left ventricular ejection fraction; NYHA, New York Heart Association functional classification for severity of breathlessness; SCD, sudden cardiac death. In the univariate and multivariate regression analysis, NYHA was correlated as a binary variable (NYHA FC I–II vs NYHA FC III–IV).

## Discussion

To our knowledge this is the first prospective study of the clinical profile, spectrum of disease-causing gene mutations and outcome in HCM from the African continent, including black Africans. Age at onset of symptoms (38.5 ± 14.3 years), male preponderance (58%), and major symptoms were similar to those reported in North American, Middle Eastern and Eastern series (Table 2).^11^,^16^,^17^ Nearly 30% of the patients bear mutations in the *MYH7* and *MYBPC3* genes, which are the commonest genetic causes of HCM.^15^

While the annual mortality rate of 2.9% was high and the overall survival of 74% at 10 years was low compared to other series of patients with HCM,^11^ the survival rate was comparable to age- and gender-matched members of the South African population. Survival was predicted by NYHA functional class at last visit.

We have found that HCM occurs predominantly in men, with a young age of onset, including black Africans, and with a positive family history of HCM in the majority. Fatigue, breathlessness and palpitations were the commonest symptoms. Atrial fibrillation was found in 9%, left ventricular outflow tract obstruction in 28%, and diastolic dysfunction in most.

In a study of the natural history of HCM in non-hospitalised Americans, Maron and others found that 55% of patients were men, the mean age was 47 years, and cardiac symptoms were present in 63% of patients.^11^ Similarly, in a study from Taiwan, Lee and colleagues found 52% HCM patients to be male, and that men had a younger age of onset of HCM compared to women.^16^ In this study, the prevalence of apical HCM was three times higher in men, and interestingly, men had a lower prevalence of LVOT obstruction. Thirty-six per cent of Taiwanese HCM patients had pulmonary oedema or paroxysmal atrial fibrillation. More recently, in the first report on the clinical characteristics of HCM in Saudi Arabia, Ahmed and co-authors found the population of HCM patients to be 71% male, and with a mean age of 42 years.^17^ Dyspnoea and palpitations were the commonest symptoms, and LVOT obstruction was found in 28%.

To date, over 1 400 mutations have been reported to cause HCM in genes encoding eight sarcomere proteins: beta-myosin heavy chain (*MYH7*), cardiac myosin-binding protein C (*MYPBC3*), cardiac troponin T (*TNNT2*), cardiac troponin I (*TNNI3*), cardiac actin (*ACTC*), alpha-tropomyosin (*TPM1*), essential light chain of myosin (MYL3) and regulatory light chain of myosin (*MYL2*).^15^,^18^ Mutations in *MYH7* and *MYPBC3* occur most often, and account for approximately 50% of HCM cases,^19^,^20^ while mutations in *TNNT2*, *TNNI3*, *ACTC*, *TPM1*, *MYL3* and *MYL2* collectively account for less than 20% of HCM cases.^21^ In our study, mutations in *MYH7* and *MYPBC3* were the commonest causes of HCM.

Moolman-Smook and colleagues have done pioneering work on the genetics of HCM in two South African sub-populations: those of European descent and those of mixed ancestry, and have previously reported on common HCM-causing mutations that arose independently and demonstrated clear founder effects in the South African population. These mutations included the *MYH7* Ala797Thr (25% prevalence),^8^ *TNNT2* Arg92Trp (15%),^9^ *MYH7* Arg403Trp (5%),^7^ *MYH7* Arg717Gln and the *MYH7* Glu499Lys^10^ mutations, which collectively accounted for 47.5% of cases of HCM from the Eastern and Western Cape provinces of South Africa. To save money and to improve efficiency, a strategy was proposed to first screen for these five founder mutations before undertaking an extensive molecular genetic screening for other HCM mutations in South Africa.^10^ However, in our study of 42 South African HCM patients, these founder mutations were absent.

The mutation yield of screening 15 sarcomeric and non-sarcomeric genes that are associated with HCM was relatively low in this study. Disease-causing mutations in any one of the sarcomeric protein genes are found in up to two-thirds of patients with HCM, and the yield of screening-associated causal genes ranges from 40–70%.^15^ The indications for molecular genetic testing in cardiomyopathy vary according to the yield of molecular testing, the cost of molecular analyses, and the impact of genetic testing on the medical management of the individual and the family. Given the relatively low yield of screening in this study, molecular genetic testing in Africans with HCM should probably not be carried out routinely as yet, until studies on the full spectrum of causal mutations and the impact of genetic testing on outcome are available.

In our study, the mean duration of follow up was 9.1 years, with an annual mortality rate of 2.9%. Complications included heart failure, atrial fibrillation, stroke and evolution to DCM. Myomectomy, alcohol septal ablation and heart transplantation were performed in a small number of patients; however no implantable cardioverter defibrillators (ICDs) were used. The high rates of mortality observed in our study may reflect, in part, the higher mortality rate of the South African population, as well as the skewed nature of tertiary-centre experience with many symptomatic patients.

In the USA, HCM was found to have an annual mortality rate of 1.3% and to be associated with stroke, atrial fibrillation, sudden cardiac death, congestive heart failure and the need for heart transplantation.^11^ In Taiwan, HCM was reported to have an annual mortality rate of 0.8%, and the mortality rate could be predicted by LVOT obstruction, atrial fibrillation and female gender.^16^ In Saudi Arabia, HCM had an annual mortality rate of 0.7%, with five ICDs inserted over seven years of follow up, and a single patient progressing to end-stage dilated cardiomyopathy.^17^

This study has a number of important limitations. First, the small sample size is a major weakness. This may account for the failure to detect the effect of known predictors of mortality in HCM, such as history of syncope and magnitude of left ventricular hypertrophy. Second, we screened for 15 genes that are commonly associated with HCM. However, there are several important HCM-causing mutations in other genes that were not included in our genetic panel, such as titin (*TTN*), myosin heavy chain gene (*MYH6*) and cardiac troponin C (*TNNC*). Therefore, there is a need for larger, prospective studies of HCM in Africa that encompass all the important genetic causes of the disease.

## Conclusions

We report on the first prospective investigation of the clinical characteristics, genetics and outcome of HCM in Africans. We found HCM to occur more in men, and with a younger age of onset. Major symptoms and complications were similar to those reported in North American, Middle Eastern and Asian studies. Known and novel disease-causing mutations were identified in the MYH7 and MYBPC3 genes, with a lower yield of mutation screening of about 30%, compared to the expected 40–70% found elsewhere. The mortality rate in this contemporary African HCM series was, however, higher than reported elsewhere, although comparable to age- and gender-matched members of the South African population. Survival was predicted by NYHA functional class at last visit.
